# Test of Oral Health Literacy in Adults (TOHLA): development and psychometric evaluation of a new scale

**DOI:** 10.1590/1807-3107bor-2024.vol38.0059

**Published:** 2024-07-15

**Authors:** Mohtasham GHAFFARI, Sakineh RAKHSHANDEROU, Fábio Luiz MIALHE, Yadollah MEHRABI, Ali SAFARI-MORADABADI

**Affiliations:** (a) Shahid Beheshti University of Medical Sciences, School of Public Health and Safety, Department of Public Health, Tehran, Iran.; (b) Universidade Estadual de Campinas –Unicamp, Piracicaba Dental School, Department of Health Sciences and Pediatric Dentistry, Piracicaba, SP, Brazil.; (c) Alborz University of Medical Sciences, School of Health, Department of Health Promotion and Education, Alborz, Iran.

**Keywords:** Health Literacy, Psychometrics, Surveys and Questionnaires, Adult

## Abstract

This study aimed to develop and rigorously evaluate the Test of Oral Health Literacy in Adults (TOHLA) for the Iranian population, addressing the limitations of existing oral health literacy (OHL) measurement instruments and contributing to the literature on OHL assessment. The development of the TOHLA involved a qualitative approach, which included a comprehensive literature review and semi-structured interviews with a panel of 15 experts from diverse fields and 22 Iranian adults aged 18 to 64 years. The instrument was designed with 48 items categorized into four domains: cognitive skill, communication skill, media skill, and functional skill. Content validity was established through expert input and content validation indices. Construct validity was supported by factor analysis, and concurrent validity was assessed by comparing TOHLA scores with demographic variables. Internal consistency and test-retest reliability analyses were performed to assess the instrument’s reliability. The psychometric evaluation of the TOHLA demonstrated strong content validity, construct validity, concurrent validity, internal consistency, and test-retest reliability. The instrument exhibited a high level of internal consistency, with a Cronbach’s alpha coefficient of 0.81 for the entire scale. Test-retest reliability was satisfactory, with an intraclass correlation coefficient (ICC) of 0.83. Concurrent validity analysis showed statistically significant associations between OHL scores and demographic variables, supporting the instrument’s overall performance. The TOHLA overcomes the weaknesses observed in existing instruments and offers a comprehensive tool with strong psychometric properties to assess the OHL of the Iranian adult population. Researchers, policymakers, and healthcare providers can utilize the TOHLA to address oral health challenges and enhance overall oral health outcomes among Iranian adults.

## Introduction

Oral health literacy (OHL) refers to the level at which individuals can access, comprehend, and apply general oral health information to make informed decisions about their oral well-being.^1^ Developing OHL skills can play a crucial role in diminishing oral health disparities and enhancing overall oral health. These skills encompass various aspects, such as understanding dental terminology, knowing the correct techniques for brushing and flossing one’s teeth, identifying common oral health problems, and recognizing the significance of regular dental check-ups.^2^ Numerous factors contribute to the prevalence of oral diseases in society. These encompass both external elements, such as financial constraints and limited access to services, and internal factors, including personality traits and caregiving behaviors.^3^ Research has consistently demonstrated that a crucial internal determinant influencing oral diseases within society is the level of OHL. At the individual level, a high OHL level enables individuals to proactively participate in self-care practices and preventive measures for their oral health. Furthermore, at the community level, OHL plays a pivotal role in addressing oral health disparities.^4-6^ Acknowledging the significance of OHL, the American Dental Association has highlighted that limited OHL poses a hindrance to the efficient prevention, diagnosis, and treatment of oral diseases.^7,8^

Moreover, health literacy plays a vital role as a social and structural determinant of health. Investigating its influence on health disparities can offer valuable insights into tackling societal health issues and implementing focused interventions. Gaining a deeper understanding of these matters can lead to the development of more effective strategies aimed at enhancing overall health outcomes.^9^

To address the detrimental impacts of limited OHL, it is essential to diagnose patients or individuals who may be facing OHL challenges. Additionally, evaluating the effectiveness of interventions is crucial. Thus, it becomes necessary to have an instrument that can accurately and comprehensively assess OHL levels.

Early OHL instruments were adapted from general health literacy instruments. For instance, the REALD^10^ was derived from the REALM,^11^ and the ToFHLiD^12^ was based on the ToFHLA.^13^ To the best of the researchers’ knowledge, to date, more than 20 instruments have been utilized to measure OHL, many of which rely on word recognition tests (such as short forms or rapid estimation) and reading skills. Among these instruments, only one was developed in Iran, but it was not an original creation. Actually, it was a compilation of previously available instruments such as REALD-30,^14^ REALD-99,^15^ REALM-D,^16^ OHLI,^17^ TOFHLiD,^12^ and CMOHK.^18^ This instrument was merely a combination of existing tools and was not specifically designed to assess the concept of OHL, as mentioned above.

## Rationale of the study

The rationale for conducting this study is grounded in the recognition of the crucial role that OHL plays in the context of oral health disparities and overall oral health improvement. OHL is an essential set of skills that empower individuals to access, understand, and apply oral health-related information to make informed decisions about their oral health and well-being.^9^ However, despite its acknowledged significance, the available instruments for assessing OHL have limitations, particularly when applied to diverse populations such as Iranians. In our study, we conducted a systematic review of various OHL measurement instruments, both in terms of dimensions (subscales) and psychometrics. The findings from this review revealed several weaknesses associated with these instruments. Many of them focused primarily on a rapid assessment of OHL, addressing only a limited aspect of OHL without offering a comprehensive view. Moreover, these instruments lacked unique and specific psychometrics due to their content and nature. Some instruments that incorporated more dimensions were found to be psychometrically flawed. To address these gaps and provide a more comprehensive assessment of OHL specifically tailored for the Iranian population, this study endeavors to develop and rigorously evaluate a new OHL instrument. The unique cultural and linguistic context in Iran necessitates a context-specific approach, as the existing instruments derived from general health literacy may not fully capture the intricacies of OHL in this setting. Therefore, this study aims to create a culturally appropriate and sensitive OHL tool that accurately reflects the multifaceted nature of OHL among Iranian adults. By developing this new instrument, the study seeks not only to assess OHL levels more effectively but also to contribute to the formulation and implementation of targeted interventions to improve oral health outcomes in the Iranian population. Understanding the OHL levels in this context can guide the development of educational programs and strategies to enhance OHL, leading to better oral health practices and overall oral health improvements in the community. The ultimate goal of this research is to advance the understanding of OHL among Iranian adults, identify potential areas of improvement, and provide a reliable means of diagnosing OHL issues. This comprehensive tool will also enable researchers and policymakers to evaluate the effectiveness of interventions aimed at enhancing OHL and, consequently, positively impact the oral health status of the Iranian population. By aligning with the cultural nuances and language specificity of the region, this study strives to contribute significantly to the ongoing efforts to promote oral health and reduce oral health disparities in Iran.

## Methods

### Study design

This methodological study aimed to create and validate the Test of Oral Health Literacy in Adults (TOHLA) specifically for the Iranian population. To achieve this aim, a comprehensive and multifaceted approach was employed to conceptualize OHL and develop a robust assessment instrument. The age group chosen for this research was 18-64 years, focusing on the adult population. Adults within this age range are typically responsible for their own health and oral care decisions and often face unique challenges and communication barriers related to oral health. By concentrating on this age group, the study aimed to provide a thorough assessment tool that caters to the specific needs and requirements of adult individuals in maintaining good oral health.

### Ethical approval

Ethical approval for this methodological study was obtained from the Research Ethics Committee of the School of Public Health & Neuroscience Research Centre at Shahid Beheshti University of Medical Sciences (Approval ID: IR.SBMU.PHNS.REC 1397.051). Participants were adequately informed about the study, and their consent to participate was obtained verbally before proceeding with the survey. This method of implied verbal consent was approved by the Ethical Board Committee of Shahid Beheshti University of Medical Sciences. All study procedures were conducted in strict compliance with the ethical standards outlined in the Declaration of Helsinki.

### Conceptualizing OHL: exploring dimensions and components

The process of developing the OHL instrument involved a meticulous and diverse approach, which included conducting a comprehensive review of the existing literature, gathering valuable inputs from expert interviews, and engaging with the target population. The primary objective was to gain a comprehensive understanding of the various conceptual dimensions of OHL, thereby constructing a strong and reliable instrument for the assessment of OHL.

### Test of OHL in adults

The TOHLA is a validated assessment tool designed to measure the OHL of adults aged 18 to 64 years. It consists of a series of questions and tasks that assess the individuals’ ability to understand and apply oral health-related information in various situations.

### Comprehensive TOHLA development

The initial step in the development of the TOHLA involved conducting a comprehensive literature review. The research team performed a thorough search in electronic databases, including Embase, PsycINFO, PubMed, Scopus, and Web of Sciences. In addition, they searched for relevant studies and resources from gray literature sources to ensure a comprehensive coverage. The search utilized key terms such as “oral,” ‘dental,” “health,” “literacy,” “tool,” “instrument,” “questionnaire,” “psychometric,” “validity,” and “reliability.”

The expert panel and the target population were interviewed after the literature review. This approach allowed the research team to gain insights from previous studies on OHL, identify key concepts and themes, and understand the different aspects and components associated with it. Following the literature review, the research team conducted semi-structured interviews with a panel of 15 experts from diverse fields, including health education and promotion, communication, psychology, and social dentistry. These interviews provided valuable expert perspectives, experiences, and recommendations, which played a crucial role in shaping the development of the TOHLA instrument.

To ensure the instrument’s relevance and applicability to the target population, additional interviews were conducted with 22 individuals aged 18 to 64 years. These interviews provided deeper insights into the challenges faced by the target audience, communication barriers encountered, and the specific information needed to maintain good oral health. The input from the target population helped refine and validate the TOHLA instrument, making it more reflective of real-world experiences and needs. The main focus of both the expert and target population interviews was to explore the essential skills and abilities individuals need to achieve a high level of oral health and effectively engage with the oral healthcare system.

In conclusion, the development of the TOHLA instrument was a comprehensive process that involved a literature review to gather existing knowledge and insights, followed by interviews with experts and the target population to ensure a well-rounded and relevant OHL assessment tool. This approach enabled the research team to create a robust and effective instrument for evaluating and promoting OHL in the general population (Figure).

**Figure:**
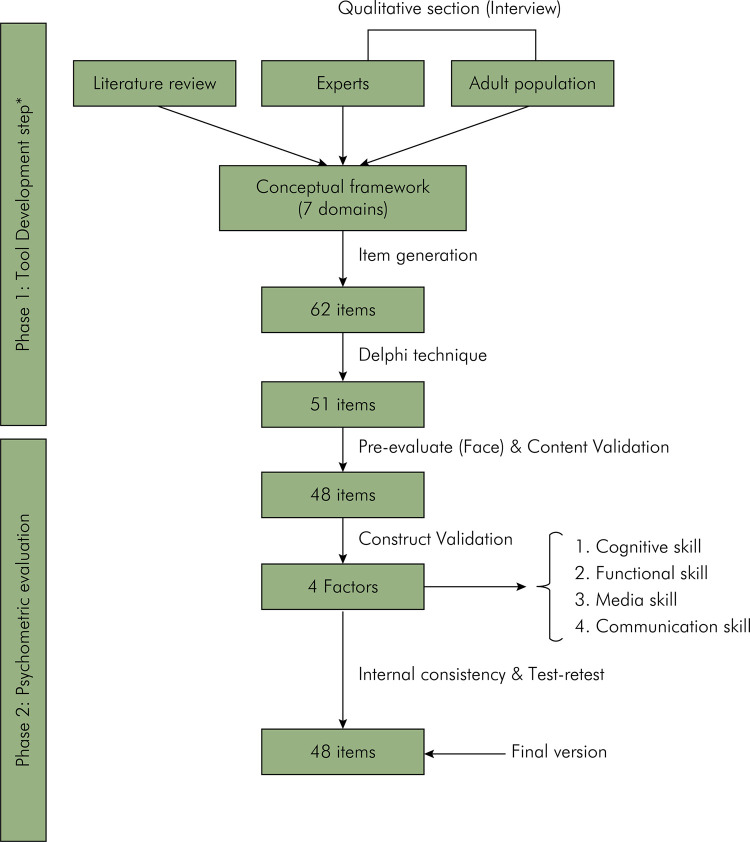
Flowchart of the development and psychometric evaluation of the TOHLA.

### Item generation

The TOHLA underwent a two-stage development (Figure). For item generation, the data were extracted from the literature review and from the interviews conducted with experts and individuals aged 18 to 64 years. The results of these interviews and the literature review were previously published by the same authors.^19^ The analysis of the findings revealed the existence of seven categories: assessment skill, emotional skill, planning skill, cognitive skill, communication skill, media skill, and functional skill. From these categories, the conceptual dimensions selected for the tool design were cognitive skill, communication skill, media skill, and functional skill. Initially, 62 items were generated during this stage, which were later refined to 48 items (the subsequent process provides further explanation).

### Delphi technique

The Delphi technique is a valuable approach employed to gather and refine expert opinions and achieve consensus on a specific subject. It involves a structured communication process that allows experts to provide their insights and feedback while remaining anonymous. The following is a more comprehensive explanation of the Delphi technique as utilized in our research: to enhance the items of our research instrument, we employed the Delphi technique to gather expert opinions and feedback. A panel of experts was carefully selected based on their knowledge, expertise, and relevant experience concerning the research topic. We recruited these experts based on recommendations from our research team and their demonstrated proficiency in the field of study, along with their professional background. Our efforts were directed at involving experts from various universities within the Tehran city area to ensure a comprehensive and diverse perspective throughout the study. In the initial round, the experts received detailed explanations of the research objectives, study scope, and the items or questions under evaluation. This information was typically conveyed through e-mail communication or an online platform. The experts were then asked to assess the relevance and importance of each item using a Likert scale, on which they rated the items numerically (e.g., 1 to 5, where 1 indicated the least relevance and 5 indicated the highest relevance). Furthermore, they were encouraged to provide comments or suggestions for improvement in a designated “Suggestions” section. The responses from the experts were collected and analyzed to identify patterns and areas of agreement or disagreement. Statistical techniques, such as calculating mean or median scores, were employed to summarize the expert ratings. Subsequent rounds followed, wherein the experts were presented with a summary of the group’s responses from the previous round, including their own ratings and the aggregated ratings of the group. This allowed the experts to reconsider their initial responses, adjust their ratings, or offer additional comments in each subsequent round. The iterative process continued until a consensus or convergence of opinions was achieved among the experts. The attainment of consensus was determined based on predefined criteria, such as a specific level of agreement or stability in the ratings across consecutive rounds. Once a consensus was reached, the final set of items or questions was determined based on the collective expert opinions.

### Scoring criteria

In this research, a comprehensive assessment of OHL was conducted using a total of 48 items. These items were classified into four distinct domains: cognitive skill (28 items), functional skill (13 items), media skill ( four items), and communication skill ( three items). To score the participants’ responses, each domain followed a simple scoring system. A correct response was assigned a score of one, while incorrect answers, “don’t know” responses, or blanks received a score of zero. Notably, within the “cognitive skill” domain, there was a table-format question with 21 subquestions. For providing 10 correct answers within this specific section, the participants were awarded 10 points. To manage the scale’s completion time effectively, each participant had 20 minutes to answer the entire set of items. This ensured a reasonable timeframe for completion.

### Pre-evaluation

The participants were recruited for the study from Tehran through convenience sampling. Before finalizing the sample, a preliminary group of the target audience, consisting of 50 individuals, answered a questionnaire as part of the pre-evaluation process.^20^ The main objective of this pre-evaluation was to gather valuable feedback on various aspects, such as the clarity and comprehensibility of the items.^20^ This step served several purposes: a) confirming the ease of following the provided instructions; b) determining the approximate time required to complete the questionnaire; and c) assessing the face validity of the questions.^21^ To evaluate face validity effectively, the researchers employed the “think out” model, in which the participants verbally expressed their thought processes while answering each item. Additionally, to enhance the evaluation further, a focus group interview was conducted involving both researchers and participants. The interview included questions such as “What do you think this section is testing?,” “Are you unfamiliar with any of the terms used in this question?,” and “Do you find this question confusing or intentionally misleading?”

Furthermore, face validity was quantitatively assessed in this study using the “impact score” method. Each item in the questionnaire was rated on a 5-point Likert scale, ranging from “completely agree” to “completely disagree,” and was then analyzed to calculate its impact score. The impact score was determined by multiplying the frequency percentage of responses by their corresponding importance. Following the calculation of the impact scores for each item, a threshold of 1.5 was set as a criterion for potential removal. Any item that obtained an impact score below this threshold was considered for removal from the questionnaire. This data-driven approach allowed for a systematic evaluation of the face validity of the questionnaire items.

It is important to note that those participants who completed the questionnaire during the pre-evaluation stage were not included in the final analysis of the interviews. Their role was solely to provide feedback on the clarity and comprehensibility of the questionnaire items.

### Psychometric evaluation

#### Participant recruitment

For the psychometric evaluation, participants were recruited using a convenience sampling method from five distinct geographical regions (North, South, East, West, and Central) within Tehran. This approach was adopted to ensure a diverse representation of the target population and simplify the recruitment process. To determine the appropriate sample size, the researchers took into account the number of items in the draft of the tool.^20,22^ With a total of 61 items in the draft, the recommended sample size was calculated to be between 5 and 10 times the number of items, resulting in an estimated target of 610 people. To account for potential dropouts or non-responses, the sample size was adjusted to 700 people.

To be eligible for participation in the study, individuals had to meet specific criteria: they needed to be 18 years old or older, residing in Iran, willing to provide a written informed consent, and literate in reading and writing. The research team utilized various communication channels to recruit participants. Potential participants were approached in person or through phone calls, e-mail messages, and social media platforms, based on their availability and preferred method of contact. This flexible approach allowed for a broader reach and increased participation. Upon expressing interest in the study, participants were provided with detailed information about the study’s purpose, procedures, and the importance of giving informed consent. Those who agreed to participate were then included in the survey.

Data collection involved participants filling out the scale through self-reporting or taking part in face-to-face interviews with the researchers, depending on their personal preference and convenience. This approach ensured that the participants could choose the data collection method they were most comfortable with. In addition to the survey data, the research team gathered demographic information, including sex, age, marital status, educational level, monthly income, employment status, residence, and smoking status. This supplementary data facilitated the comprehensive understanding of the study population and aided in analyzing the potential impact of these factors on the survey results.

## Statistical analysis

MAXQDA 2018 was utilized for the analysis of the interviews. Descriptive statistics (including mean, standard deviation (SD), and frequency distribution) were performed using SPSS 18. Additionally, psychometric evaluation using measures such as Cronbach’s alpha coefficient, intraclass correlation coefficient (ICC), and test-retest was conducted. As the data consisted of binary values (zero or one), factor analysis was carried out using the R software and Polychoric correlation matrix.

## Content validity

Content validity was assessed using two measures, namely the content validity ratio (CVR) and the content validity index (CVI). Experts were asked to evaluate each item using a three-part spectrum: “necessary,” “useful but not necessary,” and “not necessary.” To determine the minimum acceptable content validity, Lawshe’s table^20^ was consulted, with a numerical value of 0.54 set as the threshold to retain a phrase in the tool. Additionally, the Waltz & Bausell method,^23^ which considers relevance, clarity, and simplicity, was used to calculate the CVI. Any term with a score less than 0.79 was excluded from the tool.

## Construct validity

To test the construct validity, exploratory factor analysis with a varimax rotation was conducted. Prior to the analysis, the appropriateness of the data was assessed through two tests: Kaiser-Mir-Olkin (KMO) (with a threshold of < 0.6) and Bartlett sphericity (with a significance level of P > 0.05). As the data were binary (zero or one), the factor analysis was performed using the R software and Polychoric correlation matrix. The following R packages were used: “polycor,” “psych,” and “psy.”

## Concurrent validity

Concurrent validity was established by comparing TOHLA scores with demographic variables such as age, education, and income groups. It was hypothesized that older individuals and those with lower education and lower income levels would exhibit lower mean scores on the scale. Mean differences were tested using one-way ANOVA.

## Internal consistency

To assess internal consistency, the correlation level of the questions within each dimension and the total instrument in the samples related to construct validity was calculated using Cronbach’s alpha index. A Cronbach’s alpha value greater than 0.7 indicates acceptable reliability of the tool.^24^

## Test-retest reliability

The stability of the instrument was evaluated using the test-retest method, where the instrument was completed by 30 Iranian adults at two different time points, with a two-week interval between them. An acceptable correlation coefficient was set at higher than 0.75.^25^

## Results

The results in Table 1 indicate that the majority of participants fall within the age range of 25 to 45 years, accounting for 68.9% of the sample. In terms of marital status, a significant share of the participants are married (86.1%). Sex distribution shows a higher rate of females (68.1%) compared to males (31.9%). Regarding education, a considerable share hold a diploma (40.9%) or a university degree (40.4%), but 18.7% do not have a diploma. Occupational distribution reveals that freelancers make up the largest group (48.3%), followed by employed individuals (39.6%) and students (12.1%). Finally, income distribution shows that 57% of the participants earn less than 3 million (currency unit), 23.9% earn between 3 and 5 million, and 19.1% earn over 5 million.

**Table 1:** Demographic and socioeconomic characteristics of the sample population (n = 700).

Group	n	%
Age (years)		
< 25	112	16
25–45	482	68.9
> 45	106	15.1
Marital status		
Single	97	13.9
Married	603	86.1
Sex		
Male	223	31.9
Female	477	68.1
Education		
< Diploma	131	18.7
Diploma	286	40.9
University degree	283	40.4
Occupation		
Employed	277	39.6
Freelancer	338	48.3
Student	85	12.1
Income		
< 3 million	399	57
3–5 million	167	23.9
> 5 million	134	19.1

### Psychometric findings

The initial version of the instrument was developed based on the identified domains, comprising 62 items, including figures, blanks, tables, and questions. Some items were combined with the input from the research team due to their similarity in content. Eventually, 51 items from the OHL assessment section were retained for further evaluation.

### Pre-evaluation and content validity

During the pre-evaluation phase, four out of the 51 selected TOHLA instrument items were modified. Subsequently, three items were removed by content experts based on their evaluation scores (< 0.54 in the table of contents) and content validity index (CVI < 0.79). This left a total of 48 items to be examined for construct validity.

### Construct validity

To establish construct validity, the exploratory factor analysis method was employed. The KMO test was conducted on the designed questionnaire, resulting in a KMO value of 0.730, indicating the suitability of the data for factor analysis. The Bartlett test was used to examine the hypothesis that the observed correlation matrix represented a community of unrelated variables. The results of the study showed an χ2 value of 34348 with p < 0.001, supporting the presence of relationships between the variables.

The sample size used for the factor analysis was considered adequate. After conducting the factor analysis, it was confirmed that the instrument measured four underlying constructs. Table 2 presents the eigenvalues and variances of these factors. The first to fourth factors accounted for 12.72%, 8.06%, 7.13%, and 6.91% of the total variance, respectively. The minimum factor loading threshold for retaining the items in the analysis was set at 0.4.

**Table 2:** Exploring cognitive, functional, media, and communication skills: factor loading analysis.

Cognitive skill (SS loading = 12.724)					
Item	Factor load	Item	Load factor	Item	Load
A1	0.445	D2	0.843	N	0.409
A2	0.844	G1	0.579	O	0.751
A3	0.933	G2	0.891	P	0.467
A4	0.929	H	0.738	Q1	0.4323
A5	0.946	I	0.751	Q2	0.955
B1	0.470	J	0.431	R	0.424
B2	0.402	L1	0.520	S	0.935
C1	0.459	L2	0.573	W	0.465
C2	0.754	M1	0.478		
D1	0.778	M2	0.606		
Functional skill (SS loading = 6.912)					
E	0.557	X4	0.406	Y5	0.483
F	0.587	Y1	0.463	Y6	0.470
X1	0.615	Y2	0.477	Y7	0.705
X2	0.842	Y3	0.443		
X3	0.486	Y4	0.442		
Media skill (SS loading = 7.130)					
T	0.568	V2	0.955		
V1	0.937	V3	0.953		
Communication skill (SS loading = 8.069)					
K1	0.559	K2	0.518	U	0.453

### Concurrent validity

Table 3 demonstrates strong concurrent validity for the scale. The results indicate a statistically significant correlation between the research variables and the average OHL score (P < 0.05). Specifically, individuals under the age of 25 years, those with incomes above five million, as well as students and graduates (individuals with a university degree) obtained the highest average scores on the scale.

**Table 3:** Comparison of oral health literacy: mean and standard deviation analysis across demographic variables (n = 700)

Group	n	Mean ± SD	p-value
Age (in years)			
< 25	112	76.46 ± 10.51	< 0.001
25-45	482	66.90 ± 16.90	
> 45	106	61.51 ± 7.59	
(Monthly) Income			
< 3 Million	399	61.71 ± 16.11	< 0.001
3–5 Million	167	74.43 ± 10.69	
> 5 Million	134	76.69 ± 10.14	
Occupation			
Freelancer	338	67 ± 16.07	< 0.001
Employed	277	64.95 ± 14.77	
Student	85	78.72 ± 10.21	
Education level			
< Diploma	131	53.7 ± 16.14	< 0.001
Diploma	286	63.45 ± 12.61	
University degree	283	78.27 ± 9.78	

### Internal consistency and test-retest reliability

In order to assess the reliability of the instrument, both test-retest reliability and internal consistency methods were employed. The data were gathered from a sample of 30 adult residents of Tehran, and the retest was conducted in two shifts with a two-week interval between them. Additionally, a total of 700 subjects were included in the internal consistency test. The results for each domain are presented in Table 4.

**Table 4:** Reliability analysis of OHL assessment: Cronbach’s alpha and test-retest results (n = 700).

Factors	Number	Cronbach’s alpha (n = 700)	ICC (n = 30)
Cognitive skill	28	0.83	0.81 (p = 0.001)
Functional skill	13	0.77	0.76 (p = 0.004)
Media skill	4	0.85	0.79 (p = 0.002)
Communication skill	3	0.78	0.89 (p = 0.003)
Total	48	0.81	0.83 (p = 0.003)

n: Sample size; ICC: Intraclass Correlation Coefficient; p: p-value.

## Discussion

This study focused on the development and psychometric evaluation of a measurement instrument tailored to assess OHL using a qualitative approach.^19^ The aim was to create a comprehensive tool for assessing OHL among the adult population in Iran.

The results demonstrated that the designed questionnaire (TOHLA) exhibited satisfactory validity and reliability in assessing OHL within the sociocultural context of Iran.

To evaluate face validity, a qualitative approach was employed. Face validity pertains to the visual appeal and overall impression of the test, which can influence its acceptability among the target respondents.^26^

The target population of our study was highly diverse, and the ability to read and write (literacy) was used as inclusion criterion. To ensure clarity and understanding for all respondents, we designed the questions to be unambiguous, based on insights gained through a pilot study conducted in a previous publication.^19^

In assessing the reliability of our instrument, we computed Cronbach’s alpha coefficient, which yielded a value of 0.81. This value indicates an acceptable level of internal consistency for the instrument.

To further assess the consistency of scores obtained from the instrument, we employed the test-retest method with a two-week interval between assessments, following established guidelines from reliable sources.^27^

The ICC^28^for the entire instrument was determined to be 0.83, indicating a high level of internal consistency and reliability. Reliability studies suggest that a value of 0.70 is generally considered acceptable for newly developed instruments,^29^ further validating the reliability of the new instrument in question.

In the construct validation process, the KMO value was calculated to be 0.714, indicating the adequacy of the sample for factor analysis. The KMO value ranges from 0 to 1, where a score greater than 0.7 suggests that the data are suitable for factor analysis. Values less than 0.5 render the data unsuitable for factor analysis, but if they range from 0.5 to 0.69, factor analysis can still be conducted with caution.^30^ In this case, the KMO value of 0.714 confirms the appropriateness of the data for conducting factor analysis with confidence.

In our research paper, we conducted a systematic review to examine various aspects of OHL measurement instruments, including dimensions (subscales) and psychometrics. Our findings reveal notable weaknesses in these measurement instruments.^31^The existing OHL instruments in the field demonstrate numerous shortcomings, both in terms of multiple dimensions and psychometrics. Most of these instruments prioritize quick assessments of OHL, thereby addressing only limited aspects of this concept rather than providing a comprehensive view. Additionally, these instruments lack unique and specific psychometric properties, owing to their nature and content.

Furthermore, some of the OHL measurement instruments that include more dimensions are found to be flawed from a psychometric perspective.^31^ The questions designed to measure OHL with a functional perspective and skill assessment do not fully align with their intended purpose. Moreover, these instruments were primarily designed to review existing content without incorporating comprehensive expert commentary or input from the target population.

In contrast, our present instrument attempts to overcome these weaknesses, albeit partially. One of its strengths lies in employing a qualitative approach,^19^ in which we extensively reviewed the literature related to the research purpose and existing instruments. By doing so, we aimed to minimize errors during the instrument development process. However, we acknowledge that there might still be areas for improvement.

### Strengths of the study

The study boasts several significant strengths that elevate its contribution to the field of OHL assessment. Firstly, the study adopts a comprehensive approach by considering multiple dimensions of OHL, such as cognitive skills, communication skills, media skills, and functional skills. This holistic perspective ensures a thorough assessment of individuals’ OHL levels, offering a more nuanced understanding of their health literacy capacities.

Secondly, the study’s development process follows rigorous methodologies, including a comprehensive literature review, expert interviews, and the utilization of the Delphi technique. These meticulous steps contribute to the validity and reliability of the measurement instrument, instilling confidence in the accuracy of the results obtained.

Lastly, the study addresses the limitations of existing OHL measurement instruments by designing a novel and tailored tool specifically catered to the Iranian adult population. By incorporating a qualitative approach and actively seeking feedback from both experts and the target population, the instrument surpasses the shortcomings of previous tools. This process ensures that the newly developed instrument is culturally appropriate and more adept at capturing the intricacies of OHL in the Iranian context.

Not only does the combination of these strengths advance our understanding of OHL, but it also provides valuable insights for future research and practice in this domain. The study’s comprehensive nature, rigorous development process, and improvement over existing instruments contribute significantly to the advancement of OHL assessment methodologies, benefiting both researchers and healthcare practitioners alike.

## Conclusion

In this study, we successfully developed and assessed the TOHLA, a comprehensive instrument designed to measure OHL among Iranian adults. Employing a qualitative approach that involved literature review and expert interviews, we carefully crafted the TOHLA with 48 items distributed across four domains: cognitive skill, communication skill, media skill, and functional skill. The psychometric evaluation of the TOHLA demonstrated robust content validity, construct validity, concurrent validity, internal consistency, and test-retest reliability, establishing it as a reliable and consistent tool for measuring OHL. The strength of the TOHLA lies in its unique approach, which encompasses a wide range of OHL dimensions, offering a thorough and nuanced assessment of individuals’ OHL levels. Moreover, its tailored design ensures relevance and appropriateness within the Iranian sociocultural context. As researchers, policymakers, and healthcare providers seek to improve oral health outcomes and address disparities in OHL among Iranian adults, the TOHLA proves to be an invaluable instrument. By employing this comprehensive and reliable tool, stakeholders can gain deeper insights into individuals’ OHL capacities, leading to more targeted and effective interventions.

The development and validation of the TOHLA carry significant implications for the field of oral health. This comprehensive instrument, with its multidimensional approach and rigorous development process, offers a more precise and nuanced assessment of individuals’ OHL skills. By addressing the limitations observed in existing OHL measurement tools, the TOHLA elevates the standard of OHL assessment, providing valuable insights for interventions and targeted efforts to address health disparities.

The implications of the TOHLA extend across various domains. Firstly, it can greatly benefit research endeavors, enabling researchers to study the impact of OHL on oral health outcomes with a higher level of accuracy. The multidimensional nature of the TOHLA allows for a thorough understanding of the diverse factors influencing OHL, leading to more informed and insightful research findings.

Secondly, policymakers can utilize the TOHLA to design evidence-based strategies that specifically target OHL improvement. By identifying areas of low OHL proficiency, policymakers can implement tailored interventions and educational programs that address the unique needs of different populations, ultimately contributing to enhanced oral health on a broader scale. Lastly, the TOHLA holds immense potential in clinical practice. Healthcare practitioners can utilize the tool to identify patients with low OHL levels and subsequently develop personalized approaches to improve their understanding of oral health information. This tailored approach not only empowers patients to make informed decisions about their oral health but also fosters better communication between healthcare providers and patients.

Overall, the application of the TOHLA has the capacity to positively impact oral health outcomes and promote better public health. By providing a more comprehensive and accurate assessment of OHL skills, the TOHLA serves as a valuable resource for researchers, policymakers, and healthcare practitioners alike, contributing to the ongoing efforts to improve OHL and overall well-being.

Limitations of the study include the use of convenience sampling, which may introduce potential sampling bias and result in a non-representative sample, limiting the generalizability of the findings to the broader population. The large number of items (48) in the TOHLA can have implications on data collection time, respondent burden, and data processing. While the comprehensive approach enhances the assessment of OHL, the instrument’s length may lead to respondent fatigue and reduced accuracy. Future applications should consider a balance between a thorough evaluation and practicality, opting for a shorter version when necessary. Pilot studies can assess instrument feasibility, ensuring valuable insights without overwhelming participants or compromising data quality in real-world settings.
